# 3.96 kW All-Fiberized Linearly Polarized and Narrow Linewidth Fiber Laser with Near-Diffraction-Limited Beam Quality

**DOI:** 10.3390/nano12152541

**Published:** 2022-07-24

**Authors:** Shuai Ren, Pengfei Ma, Wei Li, Guangjian Wang, Yisha Chen, Jiaxin Song, Wei Liu, Pu Zhou

**Affiliations:** College of Advanced Interdisciplinary Studies, National University of Defense Technology, Changsha 410073, China; renshuai@nudt.edu.cn (S.R.); liwei06@nudt.edu.cn (W.L.); wangguangjian@nudt.edu.cn (G.W.); yishachen@hust.edu.cn (Y.C.); songjiaxin203@nudt.edu.cn (J.S.); liuwei09b@nudt.edu.cn (W.L.)

**Keywords:** high power, linearly polarized, narrow linewidth, fiber laser

## Abstract

In this paper, we realize a 3.96 kW all-fiberized and polarization-maintained (PM) amplifier with narrow linewidth and near-diffraction-limited beam quality. Based on a master oscillator power amplifier (MOPA) configuration seeded with phase-modulated single-frequency laser, a 3.96 kW signal laser is achieved with a 3 dB linewidth of 0.62 nm at the pump power of 5.02 kW. At the maximum output power, the polarization extinction ratio (PER) is ~13.9 dB, and the beam quality (M^2^ factor) is M^2^_x_ = 1.31, M^2^_y_ = 1.41. As far as we know, this is the maximum output power of PM narrow linewidth fiber laser with near-diffraction-limited beam quality and all-fiber format.

## 1. Introduction

Due to the advantages of compact structure, convenient thermal management and high electro-optic conversion efficiency, high-power narrow linewidth all-fiberized lasers with near-diffraction-limited beam quality have been widely used in the fields of spectral and coherent beam combinations [1,2,3], nonlinear frequency conversion [4], and remote communication [5]. Increasing brightness of such fiber lasers have always been the research focus. However, limited by nonlinear effects such as stimulated Brillouin scattering (SBS), stimulated Raman scattering (SRS) and thermally induced mode instability (TMI) effect [6,7,8], the increase of output power without degradation of beam quality of narrow linewidth fiber lasers still faces many challenges.

Normally, the methods of generating narrow linewidth laser seeds mainly include: narrow linewidth laser diode [9,10], multi-longitudinal-mode fiber oscillator [11,12,13], filtered superfluorescent source [14,15,16], and phase modulated single-frequency laser [17,18,19,20,21,22,23,24,25,26,27,28,29,30,31,32,33,34,35,36]. Among them, phase modulation has been proved to be a preferable method to acquire narrow linewidth high power fiber laser without obvious spectral broadening and high SRS threshold [17,18,19]. At present, sine-wave signal [25,26], pseudo-random bit sequence (PRBS) [27,28,29], and white noise signal (WNS) [30,31,32,33,34,35] have been regarded as typical phase modulation signals in fiber lasers. As for random polarization narrow linewidth fiber lasers, the output power had reached 5 kW power-level by using WNS phase modulation [31,32], and 6.12 kW output power had been realized quite recently [33]. Moreover, the narrow linewidth PM fiber lasers based on phase modulation technologies have also achieved encouraging high-power laser output. In 2016, Ma et al. adopted cascaded sinusoidal phase modulation system to realize a 1.89 kW PM fiber amplifier with linewidth of ~45 GHz, and PER of 15.5 dB [26]. In 2017, Su et al. exhibited a 2.43 kW all-PM fiber amplifier with linewidth of 0.255 nm and PER of 18.3 dB based on WNS phase modulation [34]. In 2020, Wang et al. designed a linear polarization all-fiber amplifier using cascaded WNS phase modulation, which has an output power of 2.62 kW and linewidth of 32 GHz [35]. In 2021, Wang et al. reported a 3.25 kW PM fiber laser with root mean square linewidth of ~20 GHz by using an optimized phase modulation signal [36].

Although impressive results have been obtained, the output power of PM fiber amplifiers with narrow linewidth remains at 3 kW level, which is much lower than that of non-PM ones. In fact, there are strong application requirements for all-PM narrow linewidth fiber amplifiers in the fields of coherent beam combining [2], spectral beam combining [3,37], and generation of high-power structured light beam [38]. However, the SBS gain and SRS gain in PM fibers are usually higher than those in non-PM fibers [39,40], and the TMI threshold in PM fiber lasers is significantly lower than that in non-PM ones [41]. Therefore, power scaling of narrow linewidth linearly polarized fiber lasers faces more challenges.

In this paper, a 3.96 kW all-fiberized PM narrow linewidth fiber amplifier was realized by using a bidirectional pumped configuration. The SBS effect was effectively suppressed by employing a WNS modulation system. The TMI threshold was increased by coiling the gain fiber in a racetrack spiral-shape water-cooled plate with a certain radius and using the bidirectional pumped scheme. Finally, we obtained a narrow linewidth linearly polarized fiber laser with output power of 3.96 kW. At the highest output power, the 3 dB linewidth was 0.62 nm, the PER was ~13.9 dB, and the near-diffraction-limited beam quality was M^2^_x_ = 1.31, M^2^_y_ = 1.41. No SBS effect was observed during power amplification, and the suppression ratio of SRS was about 49 dB at 3.96 kW.

## 2. Experimental Setup

**Figure 1:**
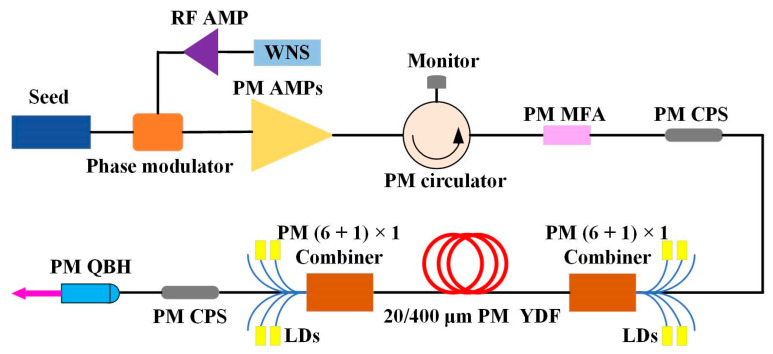
Schematic of the experimental setup. WNS, white noise signal; RF AMP, radio-frequency amplifier; PM AMPs, polarization-maintained pre-amplifiers; PM MFA, polarization-maintained mode field adaptor; PM CPS, polarization-maintained cladding power stripper; LDs, laser diodes; PM YDF, polarization-maintained Yb-doped fiber; PM QBH, polarization-maintained quartz block holder.

The experimental setup of 3.96 kW PM narrow linewidth fiber amplifier using the MOPA configuration is shown in Figure 1. The laser seed was a 50 mW, single-frequency linearly polarized, laser working at ~1064 nm. The phase modulator was driven by an amplified WNS to widen the linewidth of single frequency laser for suppressing SBS effect. Subsequently, the modulated signal was power-amplified to ~20 W by PM pre-amplifiers (PM AMPs). A PM circulator (AFR, Zhuhai, China) was used to detect the backward signal power for diagnosing the SBS effect. The pre-amplified signal entered the main amplifier through a PM mode field adaptor (PM MFA). The main amplifier adopted the bidirectional pumped configuration and used stable wavelength laser diodes (LDs) (BWT, Beijing, China) centering at 976 nm as the pump source. The pump light was injected into a double clad and large mode area PM Yb-doped fiber (PM YDF) (Self-made) with core/cladding diameter of 20/400 μm through two (6 + 1) × 1 PM beam combiners. The length of the YDF was ~8.5 m, and the absorption coefficient of the YDF was ~1.5 dB/m at 976 nm. The PM cladding power strippers (PM CPSs) at the front and rear ends of the main amplifier were employed to remove the residual cladding light in the system. Finally, the signal laser was transmitted to free-space through a PM quartz block holder (PM QBH) (Self-made).

## 3. Experimental Results

**Figure 2:**
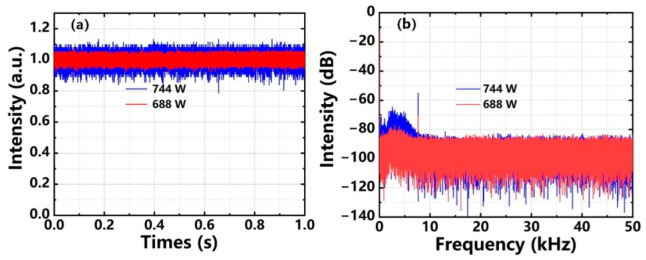
(**a**) Temporal signals of the output laser; (**b**) Corresponding PSD.

The formation of TMI was mainly due to the interaction between fundamental mode and higher order modes (HOMs), which can be effectively mitigated by increasing the relative loss of HOMs. At present, many HOMs loss methods have been proposed, among which coiling gain fiber with a certain diameter may be a simple and economical technique. Therefore, we first studied the feasibility of increasing TMI threshold by coiling gain fiber in the linearly polarized fiber amplifier using unidirectional pumped configuration. To begin with, the YDF was loosely coiled in the water-cooled plate as a circle spiral shape with a minimum diameter of 45 cm. Figure 2a,b show the temporal signals and corresponding power spectral density (PSD) of the output laser when only co-pumped power was injected. As we can see from Figure 2a,b, the temporal signal was basically stable with the standard deviation of 1.83% and the slight noise-like protuberances began to appear in the PSD when the output power was 688 W. As the output power increased to 744 W, the temporal signal demonstrated a sudden instability with the standard deviation of 3.78%, which was more than twice that at 688 W. There also existed an obvious frequency component in the PSD ranging from 0 to 10 kHz. We confirmed that the TMI threshold with loosely coiled YDF was 744 W when using the co-pumped scheme.

**Figure 3:**
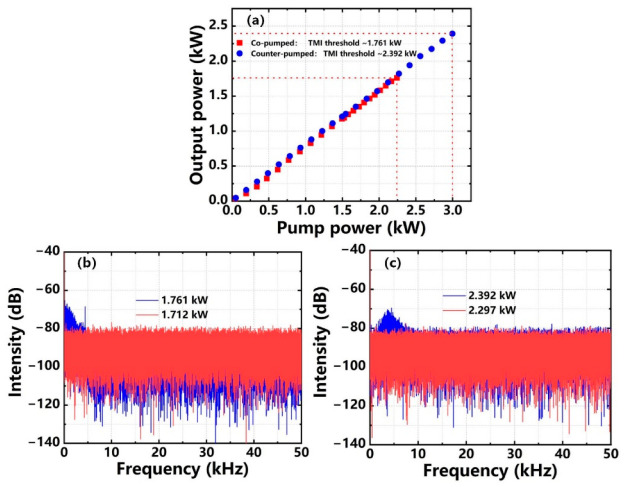
(**a**) Output power curves of unidirectional pumped scheme; (**b**) Co-pumped scheme: PSD at output power of 1.712 kW and 1.761 kW; (**c**) Counter-pumped scheme: PSD at output power of 2.297 kW and 2.392 kW.

The YDF was then tightly coiled in the water-cooled plate as a racetrack spiral shape with a minimum diameter of 8.5 cm at both ends according to our prior theoretical analysis [42]. Subsequently, co-pumped and counter-pumped schemes were used, respectively. The properties of the output laser are shown in Figure 3a–c. The red square and blue round in Figure 3a represent the output power-scaling curve with co-pumped and counter-pumped schemes, respectively. We found that the output power showed a linear growth with the increase of pump power when the unidirectional pumped scheme was applied. As shown in Figure 3b,c, when the co-pumped scheme was adopted, there existed a significantly enhanced frequency component in the PSD ranging from 0 to 10 kHz as the output power was from 1.712 kW to 1.761 kW. Based on the counter-pumped scheme, the frequency component in the PSD ranging from 0 to 10 kHz became stronger as the output power increased from 2.297 kW to 2.392 kW. Thus, the TMI thresholds of co-pumped and counter-pumped schemes were considered to be 1.761 kW and 2.392 kW, respectively. The above research proved that the TMI threshold can be remarkably increased by coiling the YDF in an appropriate way in the PM fiber amplifier. Simultaneously, theoretical research shows that, compared with unidirectional pumped configuration, bidirectional pumped configuration has more advantages in increasing the TMI threshold, and that there is an optimal proportion of counter-pumped power, which can maximize the TMI threshold of the amplifier [43]. Thus, in the following experiments, the coiling method of the YDF remained unchanged. Considering the mitigation of TMI effects, the main amplifier employed bidirectional pumped configuration, and counter-pumped power was appropriately proportioned to explore the power breakthrough ability of the PM fiber amplifier.

**Figure 4:**
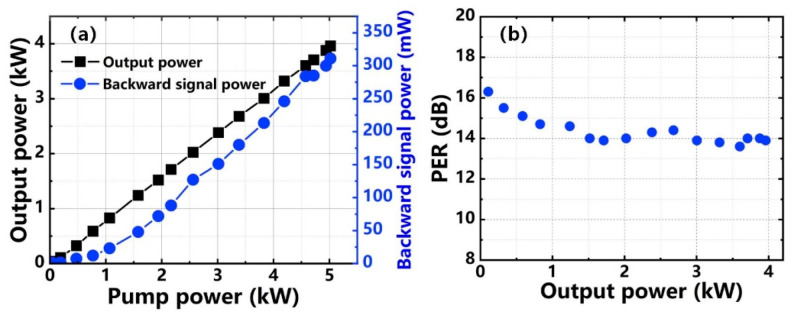
Output characteristics of the main amplifier: (**a**) Power curve; (**b**) PER.

Figure 4a,b presents the output properties of the main amplifier. As shown in Figure 4a, with the increase of pump power, the output power showed a linear growth. A record power of 3.96 kW was realized when the pump power was 5.02 kW. The slope efficiency of the amplifier was ~79.5%. At the maximum output power, the co-pumped power was 2.167 kW, and the counter-pumped power was 2.853 kW. This co-pumped and counter-pumped power distribution not only alleviated the power-handling capability of the PM combiner, but also effectively increased the TMI threshold of the amplifier. We can see from Figure 4a that the backward signal power mainly caused by the SBS effect also exhibited a linear increase trend as the amplification of the signal laser. The backward signal power at maximum output power was 311 mW, which was only 0.08‰ of the output power. This means that the SBS effect did not occur in the system during power scaling. Therefore, SBS was effectively suppressed by the WNS phase modulation. Figure 4b shows the PER of the signal laser. The PER changed between 16.3 dB and 13.8 dB during power amplification, and the PER was ~13.9 dB at 3.96 kW.

**Figure 5:**
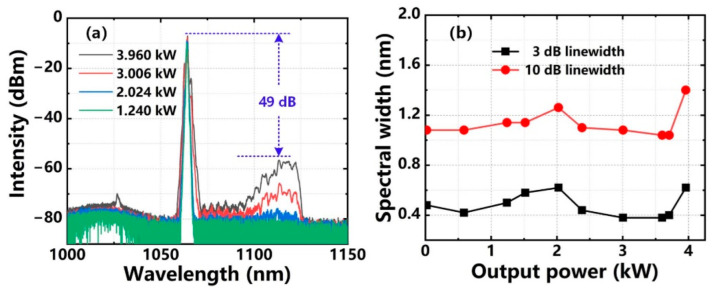
Spectral characteristics of the main amplifier: (**a**) output spectra; (**b**) 3 dB and 10 dB linewidths.

The spectral characteristics of the amplifier are shown in Figure 5a,b. Figure 5a is the spectra of signal laser at different output power. The 3 dB linewidth of signal laser at 3.96 kW was ~0.62 nm centering at ~1064.4 nm. The Raman stokes-shifted light around 1113 nm was observed at 3.006 kW, and its intensity gradually strengthened with the increase of the output power, but the signal-to-noise ratio (SNR) was as high as 49 dB compared with the spectral component around 1113 nm when the signal laser power was 3.96 kW. Therefore, SRS was effectively suppressed in our system. The evolution of the spectral linewidth of the signal laser through the power amplification was also described. As shown in Figure 5b, with the output power increase from 20 W (seed laser power) to 3.96 kW, the 3 dB linewidth of the signal laser changed from 0.48 nm to 0.62 nm, and the 10 dB linewidth of the signal laser varied from 1.08 nm to 1.40 nm. Overall, distinct spectral broadening did not occur during the power amplification.

**Figure 6:**
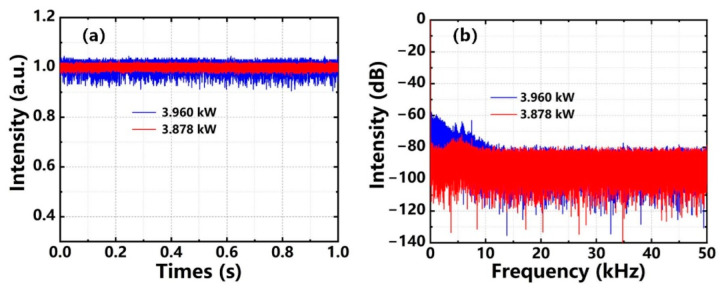
(**a**) Temporal signals of the output laser at 3.878 kW and 3.96 kW; (**b**) Corresponding PSD.

Figure 6a,b show the temporal signals and corresponding PSD of the output laser at 3.878 kW and 3.96 kW. As shown in Figure 6a, when the output power was 3.878 kW, the temporal signal of the output laser was relatively stable, but the PSD exhibited slight noise-like protuberances from 0 to 10 kHz in Figure 6b. However, the frequency component became significantly enhanced when the output power increased to 3.96 kW. At the output power of 3.878 kW and 3.96 kW, the standard deviations of the corresponding temporal signals were 0.65% and 1.78%, respectively, which was increased nearly 3 times. Therefore, the TMI effect can be considered to occur when the output power exceeds 3.878 kW.

**Figure 7:**
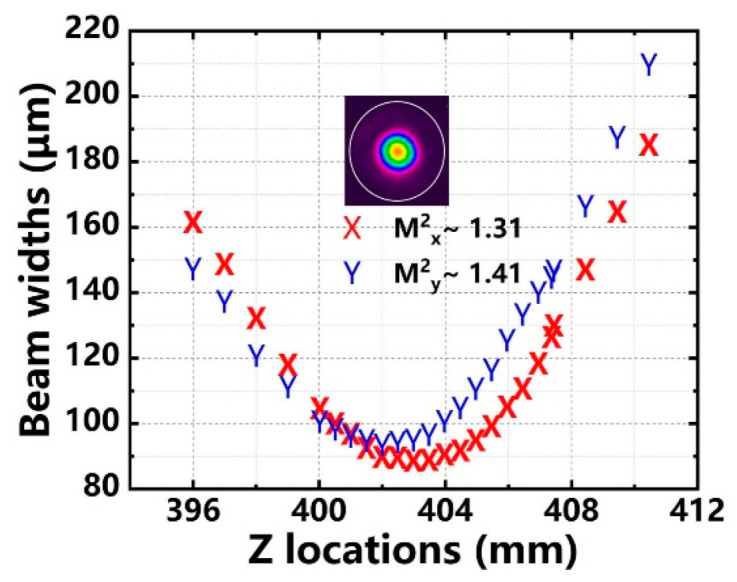
Beam quality at 3.96 kW.

Figure 7 shows the measured beam quality at the maximum output power. The M^2^ factor of 3.96 kW is M^2^_x_ = 1.31, M^2^_y_ = 1.41. That means that the PM amplifier worked at near-diffraction-limited beam quality. Meanwhile, the measured M^2^ factor of seed laser was M^2^_x_ = 1.21, M^2^_y_ = 1.33. Although the TMI was observed in the temporal signal of the output laser at 3.96 kW, there was no obvious degradation of beam quality compared with that of seed laser. The degradation of the beam quality usually lagged slightly behind that of temporal signal stability, which is common in the fiber amplifiers using gain fiber with a core/cladding diameter of 20/400 μm.

## 4. Conclusions

In conclusion, we demonstrated a 3.96 kW all-fiberized narrow linewidth PM amplifier with 3 dB linewidth of 0.62 nm based on the MOPA structure. The slope efficiency of the main amplifier was ~79.5%. The PER was ~13.9 dB, and the M^2^ factor was M^2^_x_ = 1.31, M^2^_y_ = 1.41 at the maximum output power. The backward signal power satisfied a linear growth, which revealed that there was no occurrence of SBS. SRS was observed during power scaling, but the SNR was up to 49 dB at the maximum power. Besides, the PSD of the signal laser showed obvious frequent components in the range of 0 to10 kHz at the maximum power, indicating that further power scaling is limited by TMI. New modulation signals to narrow the linewidth and TMI mitigation strategy will be the focus of future research.

## Data Availability

Data underlying the results presented in this paper are not publicly available at this time but may be obtained from the authors upon reasonable request.
